# Teachers’ Reporting Attitude Scale for Child Sexual Abuse: An Italian Adaptation

**DOI:** 10.3390/bs16020168

**Published:** 2026-01-25

**Authors:** Matteo Angelo Fabris, Claudio Longobardi, Sofia Mastrokoukou, Bruno Luiz Avelino Cardoso, Diego Costa Lima

**Affiliations:** 1 Department of Psychology, University of Turin, 10124 Torino, Italy; 2 Developmental and Educational Psychology at the Department of Human and Social Sciences, Universitas Mercatorum, 00186 Rome, Italy; 3 Laboratory of Studies and Interventions in Cognitive and Contextual Therapies, Department of Psychology, Federal University of Minas Gerais, Belo Horizonte 31270-901, MG, Brazil; 4 Department of Psychology, Minas Gerais State University, Divinópolis 35501-170, MG, Brazil; diego.lima@uemg.br

**Keywords:** teachers, child sexual abuse, reporting, psychometrics, attitudes

## Abstract

This study presents an Italian adaptation of the Teachers’ Reporting Attitude Scale for Child Sexual Abuse (TRAS-CSA), aiming to assess teachers’ attitudes towards reporting suspected cases of child sexual abuse (CSA) and to explore the scale’s psychometric properties in the Italian context. Given the critical role schools play in identifying and addressing CSA, understanding teachers’ attitudes is vital for improving reporting rates and protecting victims. A sample of 1318 Italian teachers (12.8% male; age: 25–65; *M* = 46.71; *SD* = 10.25) from various educational levels participated in the study. Exploratory factor analysis identified two primary factors: *Awareness of Role and Commitment to Reporting* (Factor 1) and *Concern and Distrust Towards Reporting* (Factor 2). Results indicated that male teachers demonstrated significantly higher scores on the concern factor, while teachers from secondary schools exhibited higher commitment to reporting compared to those from preschool and primary levels. The adapted TRAS-CSA demonstrates solid psychometric properties, providing a valuable tool for future research and intervention strategies in Italy to enhance awareness and action against child sexual abuse within educational settings. Implications for educational policies and teacher training frameworks are discussed to bolster the preventive efforts against CSA.

## 1. Introduction

Child sexual abuse is still a social emergency in several countries around the world, including Italy (Longobardi et al., 2020; Maghin et al., 2022; Stoltenborgh et al., 2011). It is estimated that between 8 and 31 percent of girls and between 3 and 17 percent of boys under the age of 18 worldwide have been sexually abused (Barth et al., 2013), and a third of these cases take place within the family (Stoltenborgh et al., 2011). A recent meta-analysis found a global lifetime prevalence of sexual harassment against children of 11%, while the prevalence of contact sexual violence was 9% (Piolanti et al., 2025). In addition, 6% of children (7% of girls and 3% of boys) reported experiencing completed forced sexual intercourse in their lifetime (Piolanti et al., 2025). However, it can be assumed that the percentage is dramatically higher, considering that a large percentage of victims tend not to disclose their victimization experiences (Brennan & McElvaney, 2020). In line with retrospective research conducted with adult samples, it is estimated that child sexual abuse is 30 times higher than official reports (Hillis et al., 2016). According to the World Health Organization (WHO, 1999), child sexual abuse (CSA) is defined as “the involvement of a child (person less than 18 years old) in sexual activity that the child does not fully comprehend, cannot give consent to, or that violates the laws or social taboos of society” (p. 15). CSA encompasses a broad spectrum of behaviors, including non-contact sexual abuse, contact sexual abuse, and penetrative acts. Extensive research has demonstrated that CSA has both immediate and long-term detrimental effects on children’s psychological adjustment, often resulting in heightened psychological distress and impairments in social, emotional, and relational functioning (Ali et al., 2024; Hailes et al., 2019; Longobardi et al., 2022). Moreover, the consequences of sexual abuse and other forms of maltreatment may persist across generations, increasing the risk of adverse outcomes for future generations (Badenes-Ribera et al., 2020). Given the profound impact of CSA on children’s survival and psychosocial development, it is essential to foster developmental environments that are sensitized to the issue of sexual violence. Such contexts should be equipped to identify warning signs and implement protective interventions to safeguard affected children (Fabris et al., 2024a).

In this regard, schools represent a highly suitable and potentially effective context for monitoring children and implementing intervention strategies for those at risk of victimization (Fabris et al., 2024a, 2024b). Indeed, school is the primary out-of-home environment where children spend the majority of their time during the developmental years and often serves as a key setting for the delivery of prevention programs (Fabris et al., 2024a). This prolonged contact enables teachers to observe signs of distress, as well as significant behavioral or emotional changes in students (Walsh et al., 2010). Moreover, from the child’s perspective, teachers may be perceived as attachment figures, fostering close and supportive relationships characterized by trust, affection, and emotional security (Fabris et al., 2022; Prino et al., 2023). Within such relational dynamics, children and adolescents may feel more confident in disclosing experiences of victimization to their teachers (Osofsky & Lieberman, 2011).

Several studies indicate that schools are among the most common sources of reports regarding suspected cases of child abuse and maltreatment (Al Azri et al., 2025; Fabris et al., 2024b). In many countries, including Italy, teachers are designated as public officials and are legally obligated to report suspected child victimization to the judicial authorities (Fabris et al., 2024b). However, despite this fundamental mandate, reporting rates often remain insufficient, and studies conducted in other countries indicate that a significant percentage of teachers (up to 11%) may choose not to report suspected cases of abuse, thereby compromising child protection efforts (Al Azri et al., 2025). This is particularly concerning, as such omissions diminish the likelihood that victims will escape ongoing abuse, thereby compromising their psychosocial well-being and allowing perpetrators to remain unpunished (Al Azri et al., 2025; Fabris et al., 2024a).

It is therefore essential to investigate the factors influencing teachers’ likelihood of reporting suspected cases of child sexual abuse. These factors include cognitive aspects (e.g., knowledge about child abuse and awareness of legal responsibilities) (Greco et al., 2020; Sainz et al., 2020), emotional variables (e.g., empathy, prosocial behavior), personal characteristics (e.g., personality traits, personal history of victimization) (Al Azri et al., 2025; Fabris et al., 2024b), and contextual elements, such as years of professional experience and perceived institutional support (Vanderfaeillie et al., 2018). Among these, attitudes toward reporting child abuse have received the most empirical attention (Al Azri et al., 2025). This construct refers to the extent to which an individual approves or disapproves of engaging in a particular behavior (Fishbein & Ajzen, 2011). Attitudes toward reporting are particularly relevant, as existing evidence indicates a strong association between such attitudes and actual reporting behaviors (Choo et al., 2013; Walsh et al., 2010, 2012a) and highlights that attitudes can be modified through training and awareness-raising initiatives (Walsh et al., 2010).

To measure *teachers’ attitudes toward CSA reporting*, Walsh et al. (2010) developed a self-report instrument based on a comprehensive and systematic literature review. The *Teachers’ Reporting Attitude Scale for Child Sexual Abuse* (*TRAS-CSA*) is a self-administered questionnaire composed of 21 items. Teachers respond using a 5-point Likert scale, indicating the extent to which they agree or disagree with each statement. In line with the *tripartite theory of attitude structure*, the scale is grounded in the notion that attitude is a multidimensional construct encompassing *affective*, *cognitive*, and *behavioral* components. The *TRAS-CSA* identifies three interrelated factors (Walsh et al., 2012b): *commitment to the reporting role*, which reflects teachers’ attitudes regarding their sense of duty and understanding of professional responsibilities; *confidence in the system’s effective response to a report*, which captures their belief that the child protection system will respond adequately and appropriately; and *concerns about reporting consequences*, which encompasses fears, anxieties, or reservations regarding the potential negative outcomes of reporting. The *TRAS-CSA* demonstrates strong psychometric properties (Walsh et al., 2012a), and shortened versions of the instrument have been adapted for use in other countries, such as Turkey (Koç et al., 2019) and Malaysia (Choo et al., 2013). However, despite the recognized psychometric characteristics of the TRAS-CSA, there is no adaptation of the tool for the Italian context. This is a limitation, not only because of the importance of teachers in combating child sexual abuse in Italy, but also because the issue of teachers’ attitudes toward CSA reporting is completely overlooked in Italy. Therefore, understanding the psychometric characteristics of this tool in the Italian context could give new impetus to research in Italy, encouraging exploration of how demographic and contextual factors influence teachers’ attitudes in Italy.

Although positive attitudes toward reporting are generally assumed to be associated with a greater likelihood of engaging in reporting behavior, the literature on this topic presents inconsistent findings (Walsh et al., 2010, 2012a). For instance, some studies indicate that stronger attitudes in favor of reporting suspected child abuse are not necessarily linked to actual reporting behavior (Goebbels et al., 2008) or even to the intention to report (Al Azri et al., 2025).

Several studies suggest that attitudes toward reporting CSA may be influenced by individual characteristics, including socio-demographic factors. In particular, available evidence indicates that greater years of teaching experience are often associated with more favorable attitudes toward reporting child abuse (Özyürek et al., 2018; Sainz et al., 2020; Walsh et al., 2012b). However, in a Malaysian sample, teachers with fewer than 10 years of experience expressed more concerns about reporting and demonstrated a stronger recognition of the value of reporting compared to their more experienced colleagues (Choo et al., 2013).

Gender has also been examined as a potential influencing factor. Some studies report that female teachers are more likely to report suspected abuse (Vanderfaeillie et al., 2018), while male teachers appear to express greater concern about the consequences of reporting (Özyürek et al., 2018). Nonetheless, other studies have failed to identify significant gender differences in teachers’ attitudes toward reporting cases of suspected victimization (Choo et al., 2013; Goebbels et al., 2008; Greco et al., 2022; Küçükaydın et al., 2023; Walsh et al., 2012b). Conversely, earlier findings suggest that male teachers may be less tolerant of child abuse—both in recognizing and reporting it (O’Toole et al., 1999)—and that male school principals are more likely to report suspected abuse consistently (Zellman, 1990).

Some studies have also explored whether attitudes toward CSA reporting vary by the educational level at which teachers work. Evidence suggests that primary school teachers (Bryant & Milsom, 2005) and primary school counselors (Sikes et al., 2010) are more likely to report suspected abuse than their counterparts in secondary education. In the Turkish context, Küçükaydın et al. (2023) found that primary school teachers demonstrated greater readiness to report CSA compared to preschool teachers. These discrepancies in research findings highlight the need for studies that take into account cultural specificities and the education system, particularly in contexts such as Italy, where there is a lack of research on teachers’ attitudes toward reporting CSA.

In summary, the existing literature underscores the critical role of teachers in identifying and reporting suspected cases of CSA, highlighting the influence of various factors, including their attitudes. While the Teachers’ Reporting Attitude Scale for Child Sexual Abuse (TRAS-CSA) has been instrumental in assessing these attitudes, research findings on demographic influences like gender, teaching experience, and educational level have been inconsistent. Crucially, a significant gap exists in understanding these dynamics within the Italian educational context, particularly concerning teachers across different school levels beyond primary and preschool. Addressing this gap is vital for developing targeted and effective prevention and intervention strategies.

### The Aim of the Study

The aim of this paper is to conduct a preliminary analysis of the psychometric characteristics of the TRAS-CSA, adapted to the Italian context, and to investigate the possible relationships between TRAS-CSA scores and some individual characteristics of teachers (gender, years of experience, school level, previous training) and the reporting of suspected cases of CSA. The literature on teachers’ attitudes towards CSA is a neglected topic in Italy. This study aims to contribute to the current state of knowledge in this particular area of research by providing data from an unexplored geographical area and also to give impetus to the Italian research community by providing it with a useful tool for future investigations. Our study focuses on CSA not only because of the profound impact on victims’ psychological adjustment and the serious violations of social taboos but also because teachers tend to perceive CSA as the most severe form of violence against a child (Walsh et al., 2012a), and because other serious forms of abuse and maltreatment are more easily detected and reported by teachers (Küçükaydın et al., 2023). Furthermore, the literature seems to have focused on primary and pre-school teachers, while teachers in service in secondary schools have only been marginally considered. This is a limitation considering that adolescents are also at risk of sexual victimization (Longobardi et al., 2021) and that Italian teachers at every school level are obliged to report cases of suspected abuse and maltreatment of minors. There is also evidence that as students get older, the number of reports made by teachers to child protection services tends to decrease (Webster et al., 2005). Overall, therefore, it seems interesting to examine teachers’ attitudes to CSA at different school levels, in addition to primary school and preschool. In Italy, children can start kindergarten at the age of 3; however, compulsory education in Italy only begins at the age of 6 with entry to primary school (Longobardi et al., 2019). After 5 years of primary school, students enter secondary school, which is divided into middle school (3 years) and high school (3 or 5 years, depending on the curriculum chosen by the student) (Longobardi et al., 2019).

## 2. Methods

### 2.1. Participants

The participants consisted of 1.318 teachers, 87.2% of whom were female. They were recruited from various school levels, ranging from kindergarten to high school. The average age of the participants was 46.71 years (*SD* = 10.25), and the average years of teaching experience was 17.65 years (SD = 11.42). Most teachers worked in public schools (89.2%), while 10.7% were in private schools. A large majority were curricular teachers (83.8%). The distribution of participants across school levels was as follows: preschool (17.9%), primary school (35.5%), middle school (21.6%), and high school (25.0%).

### 2.2. Procedure

Prior to the start of administration, the study was submitted to the university ethics committee of University of Turin, which provided its approval. The study was conducted in full compliance with Italian privacy protection and in accordance with the principles of the Declaration of Helsinki and the Italian Psychological Association (AIP). Several schools in each Italian region were contacted. For each region, we attempted to contact as many schools as possible using the public-school directories published on the Ministry of Education and Merit website. We contacted a total of 400 schools, of which 12% participated in the research. The aims of the study were explained to the school principal. Once the principal’s consent was obtained, a link to complete the online questionnaire was provided. The principal was then asked to forward the link to all the teachers and ask them to complete the questionnaire. The compilation was completely anonymous and without the possibility of tracking the device from which the teachers completed the questionnaire. Teacher participation was free and voluntary, so there was no reward. The first author was available by e-mail or telephone to all teachers who had questions about the content of the questionnaire and how to complete it.

### 2.3. Instruments

#### 2.3.1. Socio-Demographic Data

The first section of the questionnaire was aimed at capturing key sociodemographic data: age, gender, school level, years of experience, and participation in prior training on the topic of child abuse and maltreatment.

#### 2.3.2. The Italian Version of the Teachers’ Reporting Attitude Scale for Child Sexual Abuse (TRAS-CSA)

In its original version, the Teachers’ Reporting Attitude Scale for Child Sexual Abuse (TRAS-CSA; Walsh et al., 2010) is a self-report questionnaire consisting of 21 items. Teachers respond to each item on a 5-point Likert scale from 1 (strongly agree) to 5 (strongly disagree). The scale takes approximately 4 to 5 min to complete. Given the TRAS-CSA’s established presence as a widely published instrument in the scientific literature (e.g., Walsh et al., 2010, 2012a), its items and underlying structure are publicly available and routinely utilized for research and adaptation, aligning with standard academic practice. In this study, we applied a version of the TRAS-CSA adapted to the Italian context by back-translation.

#### 2.3.3. Back-Translation Procedure

An initial translation of the TRAS-CSA was conducted by the first author, a developmental psychologist with extensive experience in scientific research and clinical practice in both school and forensic child protection settings. Simultaneously, two doctoral students—both native English speakers fluent in Italian—were consulted. One student translated the scale from English into Italian, while the other back-translated it from Italian into English. These versions were compared with each other and with the independent translation by the first author. Subsequently, the translations were reviewed by the first author and three Italian researchers with expertise in developmental and educational psychology. Based on this comparative analysis, an adapted version of the instrument was finalized.

To assess the comprehensibility of the adapted version, the self-report instrument was administered to 22 university students (studying educational sciences), 12 in-service teachers from different school levels, and 1 school principal. No critical issues or specific recommendations emerged from this evaluation.

### 2.4. Data Analysis

#### 2.4.1. Exploratory Factor Analysis and Statistical Procedures for TRAS-CSA: Model Fit, Stability, and Discrimination

An Exploratory Factor Analysis (EFA) was conducted to assess the factor structure of the scale. The analysis was implemented using a polychoric matrix and the Robust Diagonally Weighted Least Squares (RDWLS) extraction method (Asparouhov & Muthen, 2010). The decision on the number of factors to retain was made using the Parallel Analysis technique with random permutation of the observed data (Timmerman & Lorenzo-Seva, 2011), and the rotation used was the Robust Promin (Ferrando & Lorenzo-Seva, 2017).

The EFA was performed using the FACTOR software (Ferrando & Lorenzo-Seva, 2017). Following modern psychometric recommendations, the model fit was evaluated through indices typically associated with Structural Equation Modeling (SEM) logic, providing an assessment of how well the model reproduces the observed correlation matrix. These indices included the Root Mean Square Error of Approximation (RMSEA), Comparative Fit Index (CFI), and Tucker–Lewis Index (TLI). According to the literature (Brown, 2006), RMSEA values should be below 0.08 with a confidence interval not reaching 0.10, and CFI and TLI values should be above 0.90 or, preferably, 0.95.

The stability of the factors was assessed using the H index (Hancock & Mueller, 2001). The H index evaluates how well a set of items represents a common factor. H values range from 0 to 1. High H values (>0.80) suggest a well-defined latent variable that is more likely to be stable in different studies. Low H values suggest a poorly defined latent variable that is probably unstable across different studies.

Finally, the item discrimination parameter and thresholds were assessed using Multidimensional Item Response Theory (MIRT) via Reckase’s parameterization (Reckase, 1985). In this framework, the discrimination parameter indicates the ability of an item to differentiate between individuals with different levels of the latent trait, while the difficulty parameter represents the level of the latent trait required for an individual to have a 50% probability of endorsing a specific response category or higher.

#### 2.4.2. *t*-Test, Normality, and Bootstrapping Procedures

A Student’s *t*-test for independent samples was conducted to investigate the extent to which teachers’ attitudes, evaluated by the two factors of the scale, differed in relation to the sociodemographic. Data normality was assessed using the Kolmogorov–Smirnov and Shapiro–Wilk tests. The assumption of homogeneity of variance was evaluated through Levene’s test. Bootstrapping procedures (1.000 resamples; 95% BCa CI) were employed to enhance the reliability of the results to correct for deviations from normality in the sample distribution and to account for differences in group sizes. Additionally, a 95% confidence interval was provided for the differences between means.

## 3. Results

### 3.1. Exploratory Factor Analysis

Bartlett’s test of sphericity (676.24; df = 91; *p* < 0.0001) and the KMO (0.861) suggested interpretability of the item correlation matrix. Parallel analysis suggested two factors as the most representative for the data (see Table 1).

Factor loadings for the items can be observed in Table 2. Composite Reliability indices and estimates of factor score replicability (H-index; Hancock & Mueller, 2001) are also reported. The items presented adequate factor loadings with high loadings in their respective factors. Only one item did not present a factor loading greater than 0.4 in any of the factors, namely item 8, which was excluded from the final version of the scale.

The fit indices of the instrument were adequate (χ^2^ = 299.792; df = 64; *p* < 0001; RMSEA = 0.055 (0.046–0.056); CFI = 0.976; TLI = 0.966). Internal consistency was confirmed by Composite Reliability (CR) and McDonald’s Omega (ω). The stability of the factor solution was assessed using Coefficient H (Hancock & Mueller, 2001). This coefficient evaluates how well a set of items represents a common factor; values above 0.80 suggest a well-defined latent variable that is likely to be stable in future studies. The item discrimination parameters and thresholds were evaluated using Item Response Theory and are presented in Table 3 and Table 4, respectively. As shown in Table 3, the most discriminative item for *Awareness of Role and Commitment to Reporting* (Factor 1) was item 5 (a = 2.130), and for *Concern and Distrust Toward Reporting* (Factor 2), it was item 10 (0.934).

Regarding the item thresholds, no unexpected response pattern was found. The higher the response category of the scale, the higher the level of latent trait required to endorse it.

### 3.2. Statistical Differences in TRAS-CSA Factor Scores

The results showed that men had a statistically higher score than women on *Concern and Distrust Toward Reporting* (Factor 2) of the TRAS-CSA. Regarding whether teachers would report a student to an authority upon suspicion, the mean for *Awareness of Role and Commitment to Reporting* (Factor 1) was statistically higher for those who answered “no” (*p* = 0.005), while for *Concern and Distrust Toward Reporting* (Factor 2), those who answered “yes” had a statistically higher mean. Furthermore, regarding *Concern and Distrust Toward Reporting* (Factor 2) of the TRAS-CSA, it was observed that teachers from secondary schools I and II had higher means than teachers from preschool and primary schools. No statistically significant differences were observed in other variables with two response options, as described in Table 5.

A Spearman correlation was conducted between years of teaching experience and the TRAS-CSA factors. The results indicated no statistically significant correlation between teaching experience and *Awareness of Role and Commitment to Reporting* (Factor 1) (ρ = 0.006; *p* = 0.836), nor between teaching experience and *Concern and Distrust Toward Reporting* (Factor 2) (ρ = 0.004; *p* = 0.872).

## 4. Discussion

The aim of our study was to investigate the psychometric properties of the Italian version of the TRAS-CSA, a self-report questionnaire designed to assess Italian teachers’ attitudes toward reporting suspected cases of sexual abuse against their students. This study is important because it provides the Italian research community with a tool that can promote empirical research in an area as complex and sensitive as the protection of minors, children, and adolescents at school. It also contributes to the development of the debate in the international literature on factors related to attitudes towards reporting child sexual abuse.

Our analysis revealed two distinct factors in the Italian context. The first factor (Factor 1) was named *Awareness of Role and Commitment to Reporting*. This factor refers to teachers’ awareness of the importance of their role in combating child sexual abuse and teachers’ determination to report cases of suspected sexual victimization against their students. The second factor (Factor 2) was named *Concern and Distrust Towards Reporting*. This factor represents a more negative dimension of attitudes toward reporting and reflects teachers’ concern (e.g., retaliation by the family or negative outcomes of child protection proceedings) and distrust in the ability of the justice system to protect a child. This last dimension could be interpreted in line with a growing sentiment among the Italian population regarding a lack of trust in the judicial system and social services. Our data do not align with the original version proposed by Walsh et al. (2010), which identified three factors.

This divergence in the factor structure from the original scale is notable and may reflect underlying cultural or systemic differences in the way Italian teachers perceive their role and the effectiveness of institutional interventions. In the Italian context, a more polarized distinction between “commitment” and “concern” may dominate reporting, as opposed to a finer division in the original three-factor model (commitment, trust, and concern). It is possible that trust in the system—originally a separate construct—merges with concerns about reporting in the Italian setting due to perceived inadequacies or mistrust. Future research should investigate whether this two-factor solution can be replicated in other samples in Italy or whether additional factors emerge in other educational contexts or regions.

We also examined some possible statistical differences in TRAS-CSA factors, but found few differences. In particular, our data show that men tended to report higher scores on factor 2 of the TRAS-CSA. This suggests that men in our sample are more likely to have concerns about reporting child sexual abuse and less confidence in the child protection system compared to women. Although the literature is not consistent on this point, our study is consistent with some previous findings that men appear to be more worried about reporting (Özyürek et al., 2018). Of course, further studies are needed to understand this finding and whether the convergence or divergence with other studies could represent, for example, a cultural aspect. 

Our data also show some significant differences in relation to school level. While high school teachers do not score differently on Factor 1 compared with elementary and preschool teachers, the data indicate that high school teachers score significantly higher on Factor 2. This finding suggests that Italian high school teachers tend to have lower confidence in the child protection system and greater concerns about reporting suspected CSA compared to elementary and preschool teachers. While the literature on teachers’ attitudes toward reporting child sexual abuse is generally sparse, it is true that it focuses primarily on preschool and elementary school teachers and only marginally considers high school teachers. In particular, no study appears to have examined high school teachers’ attitudes toward reporting sexual abuse. Some evidence suggests that elementary school staff tend to report more cases of suspected sexual abuse than secondary school teachers (Bryant & Milsom, 2005) and that the rate of reporting suspected abuse tends to decrease (Webster et al., 2005). It is possible that the nature of the interaction between students and teachers changes over time and that a more distant and less intimate relationship is observed during the high school years. This may lead students to report any adverse experiences less to their teachers and teachers therefore feeling less attentive or prepared to respond to cases of suspected sexual victimization.

Finally, we attempted to explore whether there might be a relationship between the dimensions of the TRAS-CSA and reporting behavior. We started from the assumption that teachers with a more positive attitude toward reporting are more prone to reporting behavior (Al Azri et al., 2025). However, our data revealed some unexpected results. In particular, it was found that those who reported higher scores on Factor 2 were more likely to report suspected cases of CSA. Of course, our study is cross-sectional, and we cannot say whether, for example, the experience of reporting a suspected case of CSA against one of their students had such a negative impact on these teachers that they changed their attitudes and developed more concerns about reporting or the effectiveness of child protection and safeguarding systems. At the same time, teachers who have never reported a case of suspected CSA appear to have more positive attitudes; in particular, they report higher scores for Factor 1.

This raises an important methodological consideration: the binary question “Have you ever reported?” may conflate actual reporting opportunities with a lack of reporting intent. Teachers who never encountered a suspected case would naturally not report, regardless of their attitude. Conversely, those who have reported might retrospectively adopt more critical or cautious attitudes. Future research should employ more nuanced measures, such as frequency of reporting or intention-to-report scales based on hypothetical scenarios, to disentangle these effects.

However, we must emphasize that in our protocol we only asked teachers whether they had reported a case of suspected sexual abuse of one of their students to an authority (school principal or judicial authority) during their career. However, we did not ask whether or not they decided to report a case when they had a suspicion. The wording of the question may have biased the data to some extent. In future studies in the Italian context, it would be appropriate to assess the frequency of reporting and reporting behavior specifically as a result of suspicion of a case.

No further significant difference was found in the two dimensions of the TRAS-CSA. In particular, no difference was found in our study depending on whether the teachers had received training on child abuse and maltreatment or not. However, it is crucial to highlight that this non-significant finding must be interpreted in light of the severely skewed distribution of training within our sample, where the vast majority of teachers (84%) reported having received no prior training. This finding seems to contradict what Walsh et al. (2010) suggest, indicating that training can have a profound impact on teachers by improving their awareness and attitudes toward reporting suspected cases. However, we should note that the vast majority of teachers reported that they had not received training on abuse either during their studies or during their professional careers. This situation seems to be typical of the preparation of Italian teachers on the subject, as there are no specific curricula on abuse for their training at ministerial level. Moreover, for the few teachers who reported training experiences, we are unable to determine the duration and content of the training provided and whether they specifically addressed the issue of reporting modalities and their impact, if any. This considerable imbalance significantly limits our ability to draw robust conclusions regarding the effectiveness of training on reporting attitudes within the current educational context, underscoring a pressing need for more widespread and standardized training programs.

Although we would expect greater seniority to be associated with greater aptitude for reporting suspected cases of sexual abuse, likely due to greater experience and knowledge gained, our study goes in a different direction and finds no association between years of experience and attitude for reporting CSA. In this sense, our study is not an isolated contribution, and other research has either found no association between years of experience and attitudes toward reporting suspected abuse among teachers and school personnel (Sikes et al., 2010) or found opposite results (Choo et al., 2013). Thus, it is possible that years of experience play only a minor role in attitudes toward reporting suspected CSA and that other factors may explain an indirect relationship between attitudes toward reporting and years of experience. In any case, many more studies are needed to explain these controversial results.

This study, in adapting the Teachers’ Reporting Attitude Scale for Child Sexual Abuse (TRAS-CSA) to the Italian context, has specifically elucidated the psychometric properties and factor structure of the instrument within this setting. Our analyses revealed a distinct two-factor structure—‘Awareness of Role and Commitment to Reporting’ and ‘Concern and Distrust Towards Reporting’—which provides a context-specific representation of reporting attitudes among Italian educators. This finding, along with observed variations linked to gender and school level, contributes to a more nuanced understanding of how such attitudes manifest in the Italian educational landscape. By establishing a psychometrically sound instrument tailored for Italy, this research provides a valuable tool for the Italian research community, enabling more targeted and empirically grounded investigations into teacher attitudes towards child sexual abuse reporting.

### 4.1. Limitations and Future Directions

Our study represents the first attempt to investigate the psychometric characteristics of the Italian adaptation of the TRAS-CSA. However, despite the originality of the contribution, we must consider the data obtained in the light of methodological limitations. First of all, the TRAS-CSA is a self-report instrument. This means that factors such as social desirability, memory, and text comprehension may affect the results. Therefore, future studies could use other methods (e.g., interviews) or third-party observers and could also integrate qualitative survey instruments into the protocol to capture respondents’ experiences. Another limitation is the sample. Although we collected a large sample of teachers, it cannot be considered representative of the Italian teacher population. Our sample is a convenience sample and was recruited on a voluntary basis. Furthermore, the socio-demographic data section of the questionnaire offered only two gender options (male/female). This binary choice limits the inclusivity and full representativeness of our sample and prevents a more nuanced exploration of how diverse gender identities might relate to attitudes toward CSA reporting. Future studies could invest in new research that includes representative and larger samples and offers more inclusive gender categories.

Regarding the psychometric evaluation, this study focused on an Exploratory Factor Analysis (EFA) to identify the most plausible factor structure for the Italian context. While the large sample size (N > 1300) could technically allow for a split-sample approach to perform a Confirmatory Factor Analysis, we opted to maintain the full sample to maximize the stability of parameter estimates and the accuracy of the identified two-factor solution. It is important to note that splitting a single sample does not constitute true cross-validation, which ideally requires replication in an entirely independent sample. Therefore, a CFA on a new, independent sample remains a necessary next step to verify the stability of the identified structure and allow for rigorous model comparisons with the original three-factor version.

The dimensions identified were compared with basic socio-demographic indicators, and we could not perform convergent/divergent validity as there are no similar instruments in Italy. This issue will be further developed in the future. Additionally, convergent validity could be tested by comparing TRAS-CSA scores with related constructs such as empathy, legal knowledge about reporting obligations, or measures of professional burnout. Divergent validity could be tested against unrelated constructs, such as general life satisfaction.

Finally, test–retest reliability was not considered in this study. Therefore, future studies could provide input on the reliability of the measurement of the instrument over time. Longitudinal research designs could also explore whether attitudes change over time, particularly before and after targeted training interventions. Furthermore, future studies could examine whether improvements in attitudes (as measured by the TRAS-CSA) translate into increased actual reporting behavior, helping to establish the scale’s predictive validity.

### 4.2. Practical Implications

Despite the limitations described, our study has potential practical implications. First, the adaptation of this scale to the Italian context could be an impetus for Italian researchers to develop new research on this topic and to produce new data on the psychometric properties of the instrument. Furthermore, the data at our disposal could also be useful for social and educational policies to stimulate a debate on the need to strengthen initiatives to combat and prevent child sexual abuse by involving teachers.

Specifically, this scale could be integrated into pre-service and in-service teacher training programs to assess baseline attitudes and monitor changes following educational interventions. Results may help identify teacher subgroups—such as those in secondary schools or those with less confidence in reporting mechanisms—who may benefit from tailored training modules or psychological support.

Policymakers and school administrators could use aggregated TRAS-CSA results to inform the development of standardized protocols for CSA reporting and to allocate resources for targeted awareness campaigns within the school system.

Finally, this data could also be discussed in training and awareness-raising programs on the role of the teacher in preventing and combating child victimization. By identifying specific barriers to reporting, such as distrust in the system or fear of consequences, interventions can be better aligned with real-world concerns faced by educators.

## Figures and Tables

**Table 1:** Results of parallel analysis.

Factor	Percentage of Explained Variance of Real Data	Percentage of Explained Variance of Random Data (95% CI)
1	35.15 *	17.49
2	18.25 *	15.58
3	8.59	13.67
4	6.75	12.33
5	5.64	11.10
6	5.23	9.81
7	4.39	8.69
8	4.19	7.78
9	3.42	6.85
10	3.00	5.76
11	2.16	4.67
12	2.00	3.69
13	1.24	2.37

Note. Factors 1 and 2 were retained, as their explained variance exceeds that of the random data. * indicates retained factors.

**Table 2:** Factor structure of the scale.

Items	F1	F2
1. Ho intenzione di segnalare gli abusi quando ne ho il sospetto. [*I plan to report child sexual abuse when I suspect it.*]	0.538	−0.235
2. Sarei apprensivo se dovessi denunciare un abuso su un bambino. perché temerei una ritorsione da parte dei famigliari della sospetta vittima o della comunità. [*I would be apprehensive to report child sexual abuse for fear of family/community retaliation*.]	−0.006	0.650
3. Sarei riluttante a denunciare un caso di abuso sessuale di un bambino. per paura di ciò che i genitori potrebbero fare al minore se scoprissero della denuncia. [*I would be reluctant to report a case of child sexual abuse because of what parents will do to the child if he or she is reported*]	−0.023	0.658
4. Vorrei adempire alla mia responsabilità professionale denunciando casi di abuso sessuale sui minori. [*I would like to fulfill my professional responsibility by reporting suspected cases of child sexual abuse*.]	0.778	−0.126
5. Denunciare casi di abusi sessuali sui bambini è importante per la sicurezza dei bambini. [*Reporting child sexual abuse is necessary for the safety of children*.]	0.906	0.002
6. Linee guida per la denuncia di abusi sessuali sui minori sono necessarie per i docenti. [*Child sexual abuse reporting guidelines are necessary for teachers*]	0.861	0.149
7. È importante che gli insegnanti siano coinvolti nel denunciare i casi di abusi sessuali sui minori per prevenire conseguenze a lungo termine per lo sviluppo dei bambini. [*It is important for teachers to be involved in reporting child sexual abuse to prevent long-term consequences for children.*]	0.842	0.091
9. Gli insegnanti che denunciano casi di abusi sessuali che poi non vengono comprovati. possono finire nei guai. [*Teachers who report child sexual abuse that is unsubstantiated can get into trouble*.]	0.073	0.650
10. È una perdita di tempo denunciare un abuso sessuale su un bambino perché nessuno darà seguito alla denuncia. [*It is a waste of time to report child sexual abuse because no one will follow up on the report*.]	−0.138	0.657
11. Denuncerei comunque gli abusi sessuali su un minore anche se il Dirigente Scolastico non fosse d’accordo con me. [*I would still report child sexual abuse even if my school administration disagreed with me*.]	0.487	−0.155
12. Non ho fiducia nel fatto che l’autorità risponde in modo efficace alle segnalazioni e denunce di abusi sessuali sui minori. [*I lack confidence in the authorities to respond effectively to reports of child sexual abuse*]	0.072	0.529
13. Troverei difficile denunciare un abuso sessuale su un bambino perché è difficile raccogliere abbastanza prove. [*I would find it difficult to report child sexual abuse because it is hard to gather enough evidence*.]	0.030	0.653
14. La denuncia di abusi sessuali su minori può permettere di mettere a disposizione dei bambini e delle famiglie dei servizi. [*Reporting child sexual abuse can enable services to be made available to children and families*.]	0.445	−0.079
Composite Reliability	0.874	0.801
McDonald’s Omega	0.808	0.756
H-latent	0.912	0.881
H-observed	0.935	0.887

Note. Item wording has been shortened for display purposes. Factor loadings ≥ 0.40 are typically considered meaningful.

**Table 3:** Item discrimination parameters for TRAS-CSA Items.

Items	F1	F2
1. Ho intenzione di segnalare gli abusi quando ne ho il sospetto.]	0.708	−0.309
2. Sarei apprensivo se dovessi denunciare un abuso su un bambino. perché temerei una ritorsione da parte dei famigliari della sospetta vittima o della comunità.]	−0.008	0.856
3. Sarei riluttante a denunciare un caso di abuso sessuale di un bambino. per paura di ciò che i genitori potrebbero fare al minore se scoprissero della denuncia.]	−0.031	0.881
4. Vorrei adempire alla mia responsabilità professionale denunciando casi di abuso sessuale sui minori.]	1.378	−0.224
5. Denunciare casi di abusi sessuali sui bambini è importante per la sicurezza dei bambini.]	2.130 *	0.005
6. Linee guida per la denuncia di abusi sessuali sui minori sono necessarie per i docenti.]	1.537	0.266
7. È importante che gli insegnanti siano coinvolti nel denunciare i casi di abusi sessuali sui minori per prevenire conseguenze a lungo termine per lo sviluppo dei bambini.]	1.468	0.158
8. Credo che l’attuale sistema di denuncia degli abusi sui minori sia efficace nell’affrontare il problema.]	0.132	0.015
9. Gli insegnanti che denunciano casi di abusi sessuali che poi non vengono comprovati. possono finire nei guai.]	0.094	0.838
10. È una perdita di tempo denunciare un abuso sessuale su un bambino perché nessuno darà seguito alla denuncia.]	−0.196	0.934 *
11. Denuncerei comunque gli abusi sessuali su un minore anche se il Dirigente Scolastico non fosse d’accordo con me.]	0.585	−0.186
12. Non ho fiducia nel fatto che l’autorità risponde in modo efficace alle segnalazioni e denunce di abusi sessuali sui minori.]	0.084	0.615
13. Troverei difficile denunciare un abuso sessuale su un bambino perché è difficile raccogliere abbastanza prove.]	0.040	0.854
14. La denuncia di abusi sessuali su minori può permettere di mettere a disposizione dei bambini e delle famiglie dei servizi.]	0.506	−0.090

Note. Discrimination parameters were estimated using Reckase’s parameterization. * indicates the most discriminative item for the respective factor. Item wording has been shortened for readability.

**Table 4:** Item threshold estimates for TRAS-CSA response categories.

Item No.	Threshold_1–2_	Threshold_2–3_	Threshold_3–4_	Threshold_4–5_
1	−0.160	1.517	6.253	6.253
2	−2.108	−0.746	−0.089	1.158
3	−2.498	−1.312	−0.379	0.961
4	0.332	2.641	3.895	4.682
5	1.547	4.661	5.645	6.071
6	0.738	3.123	3.968	4.513
7	0.291	2.363	3.919	4.903
8	−1.605	−0.681	0.789	1.651
9	−2.073	−0.912	0.548	1.545
10	−2.884	−2.318	−1.342	−0.105
11	−0.663	0.389	1.449	2.314
12	−1.915	−0.814	0.304	1.375
13	−2.251	−0.821	0.306	1.462
14	−0.563	1.023	2.068	2.728

Note. Thresholds represent estimated latent trait levels required to transition between response categories.

**Table 5:** Group comparisons of TRAS-CSA factors using bootstrapped independent samples *t*-tests.

	AAS		N			*t*-Test *(Bootstrapping Sample)*					
				*Mean*	*SD*	*t*	*df*	*p*	Mean Difference	CI of the Difference (95%)	
										Lower	Upper
Gender	F1	Female	1200	11.559	3.412	1.266	1357	0.185	0.364	−0.212	0.941
		Male	159	11.195	3.382						
	F2	Female	1200	20.419	4.518	−2.952	1357	0.003	−1.115	−1.792	−0.448
		Male	159	21.535	4.155						
School Level	F1	Pre-school/primary school	727	11.477	3.205	−0.440	1358	0.660	−0.819	−0.447	0.283
		Secondary school I and II	633	11.559	3.659						
	F2	Pre-school/primary school	727	20.078	4.630	−4.033	1358	0.001	−0.985	−1.464	−0.506
		Secondary school I and II	633	21.063	4.327						
Child Sexual Abuse Training	F1	No	1140	11.532	3.460	0.427	1357	0.636	0.108	−0.390	0.569
		Yes	219	11.425	3.222						
	F2	No	1140	20.516	4.419	−0.319	1357	0.769	−0.114	−0.897	−0.670
		Yes	219	20.630	4.941						
Report Abuse Authority	F1	No	1120	11.643	3.346	3.087	1361	0.005	0.746	0.224	1.230
		Yes	243	10.897	3.711						
	F2	No	1120	20.406	4.463	−2.636	1361	0.008	−0.836	−1.489	−0.118
		Yes	243	21.243	4.580						
Report Abuse School Principal	F1	No	888	11.549	3.314	0.733	1360	0.490	0.142	−0.258	0.522
		Yes	474	11.407	3.598						
	F2	No	888	20.385	4.433	−1.728	1360	0.109	−0.444	−0.948	0.068
		Yes	474	20.829	4.667						

Note. All *t*-tests use 1.000 bootstrap samples with 95% bias-corrected and accelerated confidence intervals (CI). CSA = child sexual abuse; F1 = Awareness of Role and Commitment to Reporting; F2 = Concern and Distrust Toward Reporting.

## Data Availability

The data that support the findings of this study are available from the corresponding author upon reasonable request.
